# Testing the expensive-tissue hypothesis’ prediction of inter-tissue competition using causal modelling with latent variables

**DOI:** 10.1017/ehs.2024.26

**Published:** 2024-10-14

**Authors:** Meghan Shirley Bezerra, Samuli Helle, Kiran K. Seunarine, Owen J. Arthurs, Simon Eaton, Jane E. Williams, Chris A. Clark, Jonathan C. K. Wells

**Affiliations:** 1 Great Ormond Street Institute of Child Health, University College London, London, UK; 2 INVEST Research Flagship Centre, University of Turku, Turku, Finland; 3 Department of Radiology, Great Ormond Street Hospital for Children, London, UK

**Keywords:** expensive-tissue hypothesis, human brain evolution, causal modelling, instrumental variables, body composition

## Abstract

The expensive-tissue hypothesis (ETH) posited a brain–gut trade-off to explain how humans evolved large, costly brains. Versions of the ETH interrogating gut or other body tissues have been tested in non-human animals, but not humans. We collected brain and body composition data in 70 South Asian women and used structural equation modelling with instrumental variables, an approach that handles threats to causal inference including measurement error, unmeasured confounding and reverse causality. We tested a negative, causal effect of the latent construct ‘nutritional investment in brain tissues’ (MRI-derived brain volumes) on the construct ‘nutritional investment in lean body tissues’ (organ volume and skeletal muscle). We also predicted a negative causal effect of the brain latent on fat mass. We found negative causal estimates for both brain and lean tissue (−0.41, 95% CI, −1.13, 0.23) and brain and fat (−0.56, 95% CI, −2.46, 2.28). These results, although inconclusive, are consistent with theory and prior evidence of the brain trading off with lean and fat tissues, and they are an important step in assessing empirical evidence for the ETH in humans. Analyses using larger datasets, genetic data and causal modelling are required to build on these findings and expand the evidence base.

## Graphical abstract

**Figure:**
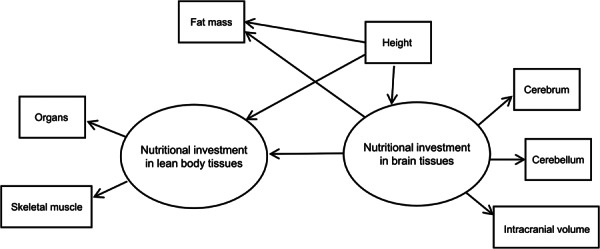

**Social media summary:** @shirleymegh et al. use causal modelling to test a version of Aiello & Wheeler's expensive-tissue hypothesis in humans.

## 1. Introduction

Upon its publication in 1995, the expensive-tissue hypothesis (ETH) broke new ground by suggesting that competition for energy resources among tissues in the body was a key consideration in questions of human brain evolution (Aiello & Wheeler, 1995). Specifically, Aiello and Wheeler posited that over evolutionary time and following shifts in dietary quality, energy from a smaller, less-specialised gut was re-allocated to fund a larger brain (Aiello & Wheeler, 1995). In the decades since, the question of how humans evolved large (Deacon, 1997), metabolically costly (Clarke & Sokoloff, 1999; Elia, 1992) brains without a compensatory increase in resting energy expenditure per kilogram body mass has continued to command great interest across disciplines. Studies in non-human animals have tested the ‘classic’ conceptualisation of the ETH (gut vs. brain), and also other formulations upholding the initial premise of the metabolically expensive brain trading off with expensive tissues like liver, skeletal muscle and testes (Isler & van Schaik, 2006; Jones & MacLarnon, 2004; Kaufman, 2003; Kotrschal et al., 2013; Liao et al., 2016; Muchlinski et al., 2018; Navarrete et al., 2011; Pitnick et al., 2006; Tsuboi et al., 2015; Warren & Iglesias, 2012). The notion that the brain might also compete for nutritional resources with less metabolically expensive tissues (i.e. those with lower mass-specific energy turnover) was introduced after empirical findings suggested a brain–fat trade-off in mammals (Navarrete et al., 2011).

The ETH as originally proposed referred to a somatic trade-off in encephalised primates, with a particular focus on humans as the most encephalised of that group. However, there are several reasons why researchers may have hesitated to test any version of the ETH in a human cohort. First, there are complications inherent to a within-, rather than across-, species analysis; namely, the difficulty of disentangling genetic from plastic or developmental effects underlying tissue/organ relationships. For example, if a brain–gut trade-off were observed in humans, would it reflect an evolutionary adaptation at the species level or the influence of developmental plasticity in individual study participants (Hales & Barker, 1992)? If both play a role, as is arguably likely, how can their differential effects be teased apart?

A second challenge comes with choosing how to operationalise variables, in other words, how to convert variables ‘from theoretical concepts into pragmatic measurable quantities’ (Smith, 2019: 594). Simple volume or mass measures of organ size, which have commonly been employed in tests of the ETH in non-human animals, may not in fact match closely to what is arguably the construct of scientific interest, namely, energetic or nutritional investment in organs and tissues. This problem was previously examined in the biological anthropology literature in the context of female energetic investment in lifetime reproduction, a variable which is similarly difficult to operationalise using demographic data alone (Helle, 2017). Although organ/tissue size is predicted to associate with nutritional investment, size alone is unlikely to perfectly, or causally, reflect the total nutritional resources allocated to a given organ or tissue (consider, e.g. costs of tissue growth and maintenance over time). The chance of any one measurement perfectly reflecting a construct of interest is small; however, the size of the gap between construct and measured variable matters when making statistical inferences. Error in defining predictor variables in an analysis will compound the more commonly recognised measurement error, resulting in causally inconsistent estimates (Becker et al., 2016; Smith, 2019).

A third problem relates to inferring the direction of causality. Although Aiello and Wheeler's original ETH did not explicitly specify a causal relationship between brain and gut size, we may reasonably wish to determine whether the size/expense of the brain drives that of the gut (or another tissue), or vice versa. (Indeed, ‘the establishment of cause and effect relationships is a fundamental objective of scientific research’; Smith, 2019: 591.) Helpfully, the ‘expensive brain’ framework (Isler & van Schaik, 2009), evidence of ‘brain sparing’ (Barker, 2004; Giussani, 2016; Hales & Barker, 1992) and allometric analyses in mammals (Smaers et al., 2021) suggest the causal direction runs from brain to body tissues; however it is very difficult to test this empirically in mammals, including humans. Relative organ size changes in hominins are posited by the ETH to have arisen over the course of hominin evolutionary history, and in general, studies of human somatic or functional trade-offs are precluded from taking an experimental approach (Helle, 2017; Smith, 2019). Reliance on observational data is often necessary, Although it introduces biases related to sampling (Henderson & Page, 2007) and both measured and unmeasured confounding variables (i.e. variables which influence both the independent and dependent variable). Biased sampling, omitted confounders and poorly operationalised traits result in independent variables correlating with the model error term. This so-called endogeneity problem violates a key assumption of regression and leads to inconsistent or even ‘completely useless’ estimates from a causal inference perspective (Antonakis et al., 2010: 1089).

In an effort to ameliorate the problems described above, the current analysis employed a structural equation modelling (SEM) approach with latent variables (see Bollen, 1989; Kline, 2023; Ullman & Bentler, 2013 for comprehensive overviews of SEM). Rather than being measured directly, latent variables are hypothesised but unmeasured constructs of scientific interest indicated by two or more measured variables. Their use helps to correct for the problem of measurement error in variables. Specifically, we used an alternative orientation to SEM which utilises model-implied instrumental variables (MIIVs) and two-stage least squares estimation (2SLS) to obtain more causally robust estimates (Bollen, 2019). Instrumental variables are secondary variables in the analysis (i.e. not themselves of scientific interest) that act as predictors of key independent variables in the model. They are assumed to be uncorrelated with the model error term and serve to ‘ensure consistency of estimates threatened by endogeneity’, including problems related to reverse causality (Antonakis et al., 2010: 1100). Instrumental variable regression is a well-established method for causal inference in fields like econometrics (Wooldridge, 2016, and see Grace, 2021 for an example in the biological sciences). However, the approach is commonly hampered by difficulties identifying proper exogenous instrumental variables. Structural equation modelling with MIIVs solves this problem by finding instrumental variables within the set of variables included in the model being tested (Bollen, 1996, 2019). As external instrumental variables were not considered during the initial design of this study, we used MIIVs to strengthen causal inference from our observational data.

Structural equation modelling with MIIVs using the 2SLS estimator (MIIV-2SLS) has several strengths over the widely used system-wide maximum likelihood estimator for SEMs, as reviewed in Bollen (2019). The use of MIIV-2SLS, for example, does not assume that model errors are normally distributed. At the same time, structural misspecifications in the measurement model introduced by incorrectly omitted paths (i.e. cross-loadings) or error covariances are less likely to be spread to other equations in the model. Similarly, MIIV-2SLS can estimate specific identified equations even if the full model is not identified (a model is ‘identified’ when a ‘unique numerical solution’ exists for each of its parameters; Ullman & Bentler, 2013: 40). Furthermore, and germane to the current analysis, small sample size may contribute to convergence problems and prevent estimation of useful parameter estimates (Bollen, 2019); MIIV-2SLS helps avoid the problem of non-convergence by being non-iterative. Finally, with MIIV-2SLS it is possible to empirically test the whole model, equation by equation, for the threat of endogeneity, therefore providing more causally reliable inferences. Four main steps to carrying out a MIIV-2SLS approach to SEM are described in the Methods section.

We employed this analytical approach in a sample of healthy participants from whom we obtained comprehensive, high-quality brain and body composition data using magnetic resonance imaging (MRI), dual-energy X-ray absorptiometry (DXA) and the four-component (4C) model of body composition assessment. Following authors who argued for extending the investigation of potential trade-offs with the brain to expensive tissues beyond the gut (Isler & van Schaik, 2006; Navarrete et al., 2011), we sought to measure the metabolically expensive liver, heart and kidneys (Elia, 1992). These organs, along with the brain, are estimated to account for 60–70% of resting energy expenditure despite making up less than 6% of body mass (Gallagher et al., 1998). The spleen, a relatively small organ, is also likely to have a high mass-specific metabolic rate (Heymsfield et al., 2012). Aiello and Wheeler (1995) argued that the gastrointestinal tract (which, with the liver, comprises the splanchnic organs) accounted for a substantial portion of resting energy expenditure, forming the basis of their argument that the gut traded off energy resources with the brain. Unfortunately, the gut is challenging to measure *in vivo* even with high-quality MRI. Without asking participants to take a drug to still movement of the gut during scanning, artefacts on MR images are difficult to avoid. For this reason, we sought to test the broader version of the hypothesis (the expensive brain trades off against other expensive organs and tissues) advanced by Isler, van Schaik and Navarette (Isler & van Schaik, 2006; Navarrete et al., 2011). These same authors introduced the notion of a brain–fat trade-off into the conversation around the ETH with their *Nature* publication in 2011. For this reason, we also measured fat mass. Finally, we measured skeletal muscle owing to published empirical evidence of a brain–muscle trade-off in primates (Muchlinski et al., 2018).

**Figure 1.**
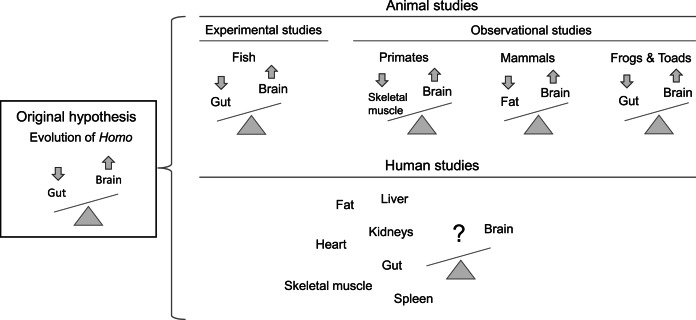
The original expensive-tissue hypothesis predicted a somatic trade-off between brain and gut size in *Homo*. Experimental and observational studies in non-human animals have tested a brain–gut trade-off or incorporated other tissues like fat and skeletal muscle (Kotrschal et al., 2013; Liao et al., 2016; Muchlinski et al., 2018; Navarrete et al., 2011). Whether there is evidence for the brain trading off with body organs or tissues in humans has remained a gap in the literature.

We set out to test the ETH's prediction of tissue competition in humans in order to fill a gap in the literature (Figure 1). With the brain and body composition data collected, we modelled two latent variables of interest – ‘nutritional investment in brain tissues’ (MRI-derived brain volumes) and ‘nutritional investment in lean body tissues’ (MRI-derived organ volume and DXA-derived skeletal muscle mass) – to test the hypothesis that the former exerts a negative, causal effect on the latter. The direction of this hypothesis follows from evidence for brain sparing (Barker, 2004; Giussani, 2016; Hales & Barker, 1992) and is consistent with the expensive brain framework (Isler & van Schaik, 2009), as noted above. Similarly, we used the brain latent and measured fat mass to test the secondary hypothesis that the brain exerts a negative, causal effect on metabolically inexpensive fat mass. All tests were performed using a single statistical model with multiple regression equations.

## 2. Methods

### 2.1. Participant recruitment and data collection

We sought to recruit 70 women of South Asian ancestry for this study. Despite overall genetic similarity, contemporary human populations demonstrate considerable variation in phenotype associated with local climatic, disease and nutritional factors, as well as historical and societal exposures. On average, body composition in South Asian populations is characterised by low levels of skeletal muscle mass and increased fat mass in relation to body size (Yajnik et al., 2003), and visceral organs also appear in South Asians to be smaller on average than in Europeans (Wells, 2016). These relative constraints in components of lean mass might represent somatic trade-offs with the brain, incorporating developmental, genetic and/or epigenetic effects (Hales & Barker, 1992; Hardikar et al., 2015; Sales et al., 2017). Assuming that smaller average body size indicates overall nutritional constraints, we predicted that negative associations between brain and body tissues may be more readily observable in South Asians than in other populations.

We included South Asian women of Indian, Pakistani, Bangladeshi or Sri Lankan ethnicity, determined by self-identification and supported by confirmation that both sets of maternal and paternal grandparents were of South Asian ethnicity. Recruitment targeted young women 20−28 years of age with a body mass index (BMI) between 17 and 28 kg/m^2^ who were healthy, nulliparous and born at term. Asian populations in general demonstrate lower average BMI compared with non-Asians (WHO Expert Consultation, 2004), hence increased adiposity or altered metabolism might occur below a BMI of 30 kg/m^2^. The upper BMI cut-off for recruitment was therefore set at 28 kg/m^2^. The designated age range, single sex and nulliparous condition were chosen to avoid phenotypic variability associated with pubertal growth, aging, sexual dimorphism and differential parity. We did not recruit women born pre-term (<37 weeks’ gestation) in order to control for the potential influence of early birth on variability in adult body composition. Individuals who reported health conditions with the potential to affect growth or metabolism, smoking or excessive alcohol use, or contraindications for MRI, were not recruited.

Posters and online advertisements were used in recruitment. Data collection took place from March 2015 to May 2016 at UCL Great Ormond Street Institute of Child Health and Great Ormond Street Hospital for Children NHS Trust, London, UK. Ethical approval was granted by the Camden and Kings Cross NHS Research Ethics Committee of the Health Research Authority (reference number 14/LO/1684). All participants gave written, informed consent.

Brain and body composition data were collected using gold standard *in vivo* techniques: MRI, DXA and the 4C model. Brain measures were the cerebrum and cerebellum, including both grey and white matter structures, and also estimated intracranial volume (all cm^3^). Body measures included fat mass, skeletal muscle mass and volumes of the heart, kidneys, liver and spleen (cm^3^). Further details on the collection of these brain and body composition outcomes are given below.

### 2.2. Anthropometry

Duplicate measures of height were taken to the nearest 0.1cm using a wall-mounted stadiometer (Holtain, Pembrokeshire, UK), with participants standing barefoot. Weight was taken in duplicate to the nearest 0.01 kg during the air-displacement plethysmography procedure (see below), with BMI calculated as weight/height^2^ (kg/m^2^).

### 2.3. Fat mass

The 4C model of body composition assessment, recognised as the gold standard *in vivo* method, was used to estimate fat mass, as described previously (Fuller et al., 1992; Wells et al., 2015). Compared with simpler models, the 4C model delineates the fat, water, protein and mineral components of body weight, thus minimising theoretical assumptions related to properties of fat-free mass to yield more robust outcomes. The measurements described below contributed data for use in the 4C model. All measurements were performed by the first author (MSB).

#### 2.3.1. Body volume

Body volume was measured by air-displacement plethysmography using the BodPod system (Cosmed, Rome, Italy). Participants wore minimal, tight-fitting clothing and a swimming cap during the procedure to avoid detrimental effects on measurement accuracy of air trapped within hair and loose clothing (Dempster & Aitkens, 1995). Two tests comprising a minimum of four body volume measurements were carried out for each participant. Age- and sex-specific equations were used to predict lung volume (Wells et al., 2012), and raw body volume was adjusted for thoracic gas volume and surface area artefact (warmer, more compressible air near the skin surface; Dempster & Aitkens, 1995). The precision of body volume measurement in our lab is 0.24 l (Wells et al., 2012).

#### 2.3.2. Body water

Total body water was measured by deuterated water isotope dilution. An oral dose equivalent to 0.05 g of deuterated water per kg body weight (99.9 atom% deuterium, Sigma Chemical Co., Poole, UK) was administered to participants following the collection of a baseline saliva sample using an absorbent salivette (Sarstedt, Rommelsdorf, Germany). A post-dose saliva sample was collected following a 4 h equilibration period. Isotopic enrichment of the pre-, post-dose, and dose samples was measured using isotope-ratio mass spectrometry (Thermo Delta XP with Gasbench). Raw total body water was adjusted for potential overestimation of the dilution space, and for liquids consumed during the equilibration period (Williams et al., 2006). The precision of this measurement in our lab is 1% (Wells et al., 2012).

#### 2.3.3. Bone mineral content

Bone mineral content was assessed by DXA (Lunar Prodigy, Madison, WI, USA), with participants wearing light clothing and no removable metal to avoid artefact on scan images. One whole-body scan of approximately 5 min duration was performed with participants lying supine on the scanner bed. The estimated radiation exposure is 2 μSv, lower than daily background exposure in the UK. Bone mineral content was derived directly from the scanner software (Encore, version 14.10.022). The precision of bone mineral quantification by this method is reported to be 1.1% (Kiebzak et al., 2000).

Fat mass was calculated with the following equation (Fuller et al., 1992):
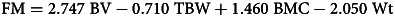
where BV is body volume (l), TBW is total body water (l), BMC is bone mineral content (kg) and Wt is body weight (kg).

### 2.4. Skeletal muscle mass

Skeletal muscle mass was estimated by DXA. Data on appendicular lean tissue (summed arms and legs) were derived following the whole-body DXA scan described above. A single observer (MSB) isolated the limbs from the trunk using regional DXA software-generated lines with manual adjustment for variability in body size/shape. Arms were defined as the area extending from a line drawn through the humeral–glenoid juncture to the tips of the phalanges. Legs were defined as the area extending from a line drawn through and perpendicular to the femoral neck to the phalange tips (Szulc et al., 2004). Appendicular lean soft tissue can be used as a proxy for whole-body skeletal muscle mass as it is closely correlated with skeletal muscle estimated using CT and MRI (Kim et al., 2002).

### 2.5. Brain and body organ volumes

High-resolution 3D imaging of the brain, chest and abdomen was undertaken using a 3T Siemens Magnetom Prisma scanner (Siemens, Erlangen, Germany). The following sequences were acquired: a T1-weighted MPRAGE sequence for brain volume (TR = 2000 ms, TE = 2.74 ms, flip angle = 8°, voxel size = 1 mm^3^ isotropic, slices = 240, duration = 5 min); a T2-weighted, turbo spin echo SPACE sequence for the abdomen (TR = 2000 ms, TE = 220 ms, flip angle = variable, voxel size = 1.5 mm^3^ isotropic, slices = 144, duration = 7 min); and for the chest, a T2-weighted TrueFISP sequence with breath-hold (TR = 475 ms, TE = 1.53 ms, flip angle = 47°, voxel size = 1.5 × 1.5 × 4.0 mm, gap = 0, slices = 42, duration = 20 s).

The T1-weighted MR images were processed and segmented with FreeSurfer (version 5.3; Fischl et al., 2004) to derive cerebral and cerebellar grey and white matter brain volumes. Estimated total intracranial volume, an estimate of the volume of the cranium, is included in FreeSurfer's standard data output (https://surfer.nmr.mgh.harvard.edu/fswiki/eTIV, and see Buckner et al., 2004). The heart, kidneys, liver and spleen were manually segmented by a single observer (MSB) from raw MRI data using the open-source OsiriX DICOM viewer (version 8.5; Rosset et al., 2004). Regions of interest were drawn around organs in contiguous image slices. The software automatically calculated organ volume by summing the regions of interest and multiplying by the slice thickness in isotropic datasets. Duplicate organ volumes were derived on different days and averaged. The technical error of measurement for duplicate measures was 1.9% for the heart, 1.1% for the left kidney, 0.7% for the right kidney, 0.7% for the liver and 1.4% for the spleen. Preliminary analysis suggested that heart volume was overestimated, thus this outcome was adjusted using a literature-based value (Prodhomme et al., 2012; Shirley et al., 2019).

### 2.6. Statistical analyses

Data were plotted for visual inspection, with normality of variables assessed using histograms and the Shapiro Wilk test. Mean, standard deviation and range were reported for raw continuous data. Coefficients of variation were also reported. Pearson correlations were used to examine relationships among the variables. These and the analyses described below were carried out in R version 4.2.2 (R Core Team, 2022).

#### 2.6.1. Structural equation modelling with latent variables

**Figure 2.**
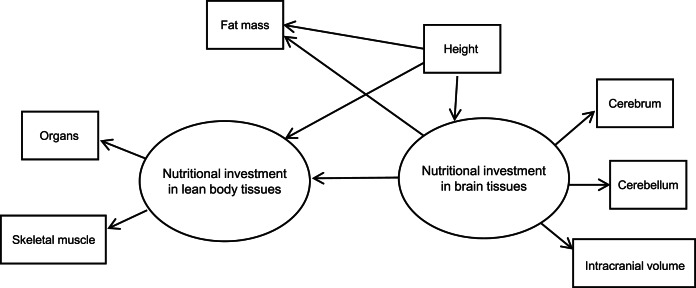
Basic structural equation model with multiple-indicator latent variables. ‘Nutritional investment in lean body tissues’ is measured by organ volumes (heart, kidneys, liver spleen) as a composite variable, and skeletal muscle mass. ‘Nutritional investment in brain tissues’ is measured by cerebrum, cerebellum and intracranial volumes. Observed variables are denoted with rectangles and unobserved latent constructs with ovals. Single-headed arrows pointing from latent variables to observed variables (skeletal muscle, organs, cerebrum, cerebellum and intracranial volume) denote factor loadings, while those pointing at observed or unobserved response variables (i.e. the two latent constructs and fat mass) represent structural (causal) path coefficients. Symbols for different model parameters (i.e. intercepts, factor loadings, disturbances and path coefficients) are not included in the diagram for simplicity.

We used structural equation modelling with multiple-indicator latent variables to model the hypothesised causal effect of brain on body tissues in humans. Our basic SEM showing model components and hypothesised relationships is shown in Figure 2. The model assumes two theoretical (unobserved) latent constructs, denoted by ovals. These are ‘nutritional investment in lean body tissues’ and ‘nutritional investment in brain tissues’. The former is assumed in the model to causally affect two measured (observed) variables, which are denoted by rectangles: organs (summed heart, liver, kidney and spleen volumes) and skeletal muscle (in kilograms).

The brain latent, in turn, is assumed to causally affect measured cerebrum, cerebellum and intracranial volumes. These measured indicator variables allow us to tap into the brain and body tissue constructs of true interest (i.e. energetic/nutritional investment in tissues and organs), which are themselves impossible to measure directly, or with great accuracy, using our dataset (Helle, 2017; Ullman & Bentler, 2013). Importantly, the latent constructs are free of measurement error contained in the observed variables that indicate them (Bollen, 1989).

Our model in Figure 2 has two parts. First, the measurement model describes how a given latent variable is ‘measured’ by observed variables in the dataset. For example, the equations for our lean body latent construct with two observed variables (organs and skeletal muscle) look like:
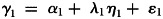

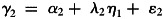
where *η* is the latent variable that is assumed to causally affect *γ* (the observed indicator variable), *α* is the intercept and *λ* is the parameter indexing the change in a given measured indicator variable for a one-unit change in the latent variable (i.e. its loading). As a theoretical construct, the latent variable does not have an inherent unit of measurement, but the scale can be set by designating a measured indicator variable with a fixed, unstandardised loading of 1 (Bollen, 1989; Hayduk & Littvay, 2012). The *ɛ* in the equations above represents unique error terms for the indicator variables, considered to be uncorrelated with *η* and with one another (Bollen, 1989). In our model, the constructs ‘nutritional investment in lean body tissues’ and ‘nutritional investment in brain tissues’ and their indicator variables make up the measurement model. Indicator variables intracranial volume, organs, cerebrum and cerebellum, in addition to height, were rescaled by factors of 10 or 100 to reduce disparities in variance. Measured variables intracranial and organ volumes were used to set the scale for the brain and lean body latents, respectively. Both constructs were modelled as continuous latent variables.

The second part of the SEM is the latent variable model (also commonly referred to as the structural model, although Bollen (1989, 2019) has advised against using this terminology). The latent variable portion of our model includes an equation hypothesising a negative, causal effect of ‘nutritional investment in brain tissues’ on ‘nutritional investment in lean body tissues’. By specifying this model, we are engaging in a confirmatory analysis based upon the inference that nutritional investment in brain tissues is negatively, causally related to nutritional investment in body tissues, as indicated by the direction of the arrow pointing from the brain to body latent.

As a secondary hypothesis, we included measured fat mass in the model to test whether the brain tissue latent variable exerted a negative, causal effect on fat mass. Our rationale for analysing fat and lean body tissues separately was based on the fact that (1) fat is relatively more plastic than lean mass (i.e. more variable over the life course, with the ability to change quickly over relatively short time periods) and relatively metabolically inexpensive, (2) unlike other tissues in the body, fat serves to store energy that can be used (now or in the future) to fund a range of bodily functions including those involving other tissues (Wells, 2012), and (3) fat and brain tissue have been suggested by theory and empirical findings to represent ‘alternative solutions’ to ecological stresses (see Discussion). Measured height was included in the model to adjust for body size (i.e. allometry), seen in the model as an observed variable predicting the two latent constructs and measured fat mass. Although body mass is commonly used to scale analyses of metabolic rates across species, we opted to use height as it is arguably a better, more stable reflection of adult body size in our human cohort (i.e. less variable and less susceptible to fluctuation via changes in fat mass).

A main strength of SEM is its ability to combine latent variable and measurement models with simultaneous equations. This allows for the estimation and removal of measurement error, thus freeing latent variable parameter estimates from bias (Bollen, 1989; Ullman & Bentler, 2013).

#### 2.6.2. Model-implied instrumental variable approach to SEM

Instrumental variables are secondary variables, not themselves of scientific interest in the analysis, that act as predictors of key independent variables in the model from which causal inference is made. The MIIV-2SLS approach differs from the classical system-wide maximum likelihood-based SEM by estimating the model equation-by-equation instead (Bollen, 1995, 1996, 2019). This is beneficial in terms of robustness to model misspecification and its diagnostics (i.e. local tests of model fit), distributional assumptions, model convergence and small sample performance (Maydeu-Olivares et al., 2019). The MIIV-2SLS approach is based on four main steps.

First, as in regular SEM, one specifies the model. In the second step of the MIIV-2SLS method, latent variables are transformed to observed variables by replacing them with their respective scaling indicators, minus their errors. Original intercepts and factor loadings are conserved. Third, instrumental variables are found among the observed variables already included in the model with the following general instrumental variable properties: they are (1) uncorrelated with the equation error, but (2) ‘sufficiently correlated with the problematic endogenous explanatory variables that correlate with the error’ (Bollen, 2019: 35). Owing to our moderate sample size, we did not use all the MIIVs available for these data. Instead, two more than needed for identification were selected from those model-implied instrumental variables that led to the largest increment in model *R*^2^ from the first-stage regression; this strategy has been shown to produce the most reliable results (Bollen et al., 2007). Further details of how to find MIIVs are given in Bollen (2019).

At step four, the model is estimated with two-stage least squares. If there is more than one MIIV per explanatory variable, the equation is overidentified. Sargan's test can then be used to assess whether the equation-specific MIIVs are uncorrelated with a given equation's error, which is an essential assumption of the MIIV method. The *p*-values given in the Sargan's test results help to diagnose which equations are inconsistent with the data; higher *p*-values mean one cannot reject the hypothesis that the MIIVs are uncorrelated with the equation error. That is, if no Sargan's tests are statistically significant, one can conclude that the overall model fits the data and make causal inferences from the estimated parameters (Antonakis et al., 2010).

All steps described above are automated using the R package MIIVsem (version 0.5.8; https://cran.stat.unipd.it/web/packages/MIIVsem/MIIVsem.pdf; Fisher et al., 2017). The following arguments were set within the package's miive function: sarg.adjust = ‘holm’, se = ‘boot’, bootstrap = 5000, boot.ci = ‘bca’, missing = ‘twostage’, and overid.degree = 2. The overid.degree argument specifies that two more MIIVs than needed for identification should be selected, as described above. Standard errors were computed using a nonparametric bias-corrected and accelerated bootstrap assuming an independent random sample, based on the standard deviation of 5000 successful bootstrap replications. The sarg.adjust argument adjusts *p*-values associated with the Sargan test for multiple comparisons using the method of Holm (1979). Three missing observations for the organs variable were handled with MIIVsem's two-stage procedure, where, after saturated population means and covariances are calculated, these are then used to calculate the MIIV-2SLS structural coefficients in the second step (see page 14 of https://cran.stat.unipd.it/web/packages/MIIVsem/MIIVsem.pdf for further details). Importantly, MIIV-2SLS treats factor variances and indicator residual variances as nuisance parameters; therefore, these parameters are not included in MIIV equations (Bollen et al., 2022). Finally, we conducted a sensitivity analysis, running the model including just the *n* = 67 with complete data.

## 3. Results

A convenience sample of 70 participants was recruited in London, UK. Descriptive statistics are given in Table 1. Mean age was 24 years and there were wide ranges for weight (40.7–81.1 kg) and height (147.8–177.3 cm), with means of 57.8 kg and 161 cm, respectively. Weight was four times more variable, with a coefficient of variation of 16.1 vs. 4.1% for height. Mean BMI was 22.3 kg/m^2^. One participant with a BMI of 30.3 kg/m^2^ was recruited; everyone else fell within the target range of 17–28 kg/m^2^. Mean ± standard deviation, range and coefficient of variation are also given for all body organs, tissues and brain components measured. There were very few missing data.

**Table 1.** Descriptive statistics for the sample

Subject characteristic	*n*	Mean ± SD	Range	Coefficient of variation (%)
Age (years)	70	24 ± 2.4	20–28	—
Weight (kg)	70	57.8 ± 9.3	40.7–81.1	16.1
Height (cm)	70	161 ± 6.6	147.8–177.3	4.1
BMI (kg/m^2^)	70	22.3 ± 3.5	17.2–30.3	15.6
Heart (cm^3^)	69	387 ± 69	256–605	17.8
Liver (cm^3^)	70	1140 ± 202	722–1599	17.7
Kidneys (cm^3^)	70	277 ± 48	197–453	17.5
Spleen (cm^3^)	68	132 ± 46	73–310	34.9
Organs (cm^3^) (composite)	67	2055 ± 327	1410–2811	15.9
Cerebrum (cm^3^)	70	868 ± 72	683–1019	8.3
Cerebellum (cm^3^)	70	127 ± 11	102–154	8.7
Brain (cm^3^) (composite)	70	1041 ± 78	851–1210	7.5
Fat-free mass (kg)	70	37.6 ± 4.3	28.4–48.8	11.4
Fat mass (kg)	70	20.3 ± 6.7	8.3–40.1	32.9
Skeletal muscle mass (kg)	70	15.3 ± 2.2	10.8–20.2	14.2
Estimated total intracranial volume (cm^3^)	70	1293 ± 102	1061–1541	7.9

**Figure 3.**
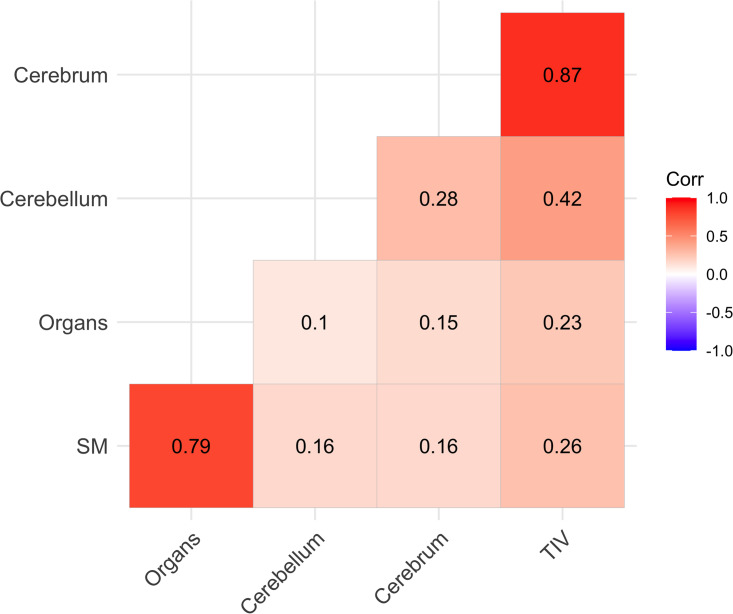
Pearson correlations among brain and body outcomes used as indicator variables. The topmost three boxes show correlations among brain latent indicator variables, while the bottom leftmost box shows the correlation between the two body latent indicator variables. SM is skeletal muscle; TIV is estimated total intracranial volume; and the organs variable is the summed volumes of heart, liver, kidneys and spleen.

Figure 3 shows correlations among the indicator variables used to measure latent constructs; moderate-to-high correlations are expected for indicators theoretically caused by the same underlying construct (Kline, 2023). We see particularly strong correlations for SM with organs and for cerebrum with intracranial volume.

The results of the SEM are given in Table 2. With respect to the measurement model, all indicator variables loaded significantly and positively onto the brain and lean body tissue latent variables. Sargan's test statistics were not significant, consistent with the null hypothesis that MIIVs are uncorrelated with equation error. This supports a causal interpretation of our estimates by confirming the appropriateness of both measurement and latent variable parts of the model. In the latent variable part of the model, adjusting for body size, we found a negative causal estimate for the regression of the lean body tissue latent on the brain latent (−0.41); however estimates ranging from a larger negative value to a small positive value (95% CI, −1.13, 0.23) were also compatible with the data under our model. We similarly found a negative, causal estimate for measured fat mass on the brain latent (−0.56), although with a much wider confidence interval indicating more uncertainty in the estimate (95% CI, −2.46, 2.28).

**Table 2.** Model-implied instrumental variables with two-stage least squares estimation (MIIV-2SLS) estimates for latent variable and measurement models testing negative causal effects of the latent variable ‘nutritional investment in brain tissues’ on the latent ‘nutritional investment in lean body tissues’, and of the brain latent on measured fat mass (*n* = 70)

Measurement model	Coefficients				Sargan test^b^		
	Estimate	Standard error^a^	Lower CI	Upper CI	Statistic	d.f.	*p*
Body measured by: Organs Skeletal muscle	1.00 0.60	0.07	0.47	0.74	1.02	2	1.00
Brain measured by: Intracranial volume Cerebrum Cerebellum	1.00 6.03 0.39	0.85 0.12	4.06 0.16	7.45 0.65	4.47 2.03	2 2	0.53 1.00
*Latent variable model*							
Body regressed on: Height Brain	2.79 −0.41	0.52 0.35	1.82 −1.13	3.85 0.23	0.02	1	1.00
Brain regressed on: Height	0.55	0.17	0.22	0.86			
Fat mass regressed on: Height Brain	1.20 −0.56	1.32 1.17	−1.28 −2.46	3.84 2.28	1.87	1	0.69
Intercepts: Body Brain Cerebellum Cerebrum Fat mass Skeletal muscle	−19.25 4.11 7.72 8.73 8.20 3.06	7.72 2.65 1.59 11.01 19.69 1.46	−33.77 −1.01 4.33 −9.68 −31.44 0.13	−3.17 9.38 10.67 33.93 46.85 5.83			

a Bootstrapped standard errors based on 5000 repetitions

b Sargan tests for latent variable and measurement model equations test the hypothesis that MIIVs are uncorrelated with equation error; *p* > 0.05 indicates failure to reject the null hypothesis of no correlation.

CI, confidence interval.

Using listwise deletion for missing data instead of MIIV SEM's two-stage procedure revealed results consistent with those from the larger sample. Causal estimates for the body latent regressed on the brain latent, and for measured fat mass regressed on the brain latent, in both cases controlling for height, were reduced in size but remained negative (Supplementary Table S1). However, had we used traditional SEM without model-implied instrumental variables and assumed, but not actually tested, for the lack of endogeneity problems as done in MIIV-2SLS, the conclusion would have been the opposite: we ran such a model, and it suggested a small, positive estimate for the regression of the body latent on brain latent (0.13, 95% CI, −0.52, 0.78); Supplementary Table S2).

## 4. Discussion

Our findings, based on a state-of-the-art causal modelling technique, have filled a gap in the literature by testing the ETH's prediction of tissue competition in humans for the first time. Specifically, we predicted a negative causal relationship between the latent variables ‘nutritional investment in brain tissues’ and ‘nutritional investment in lean body tissues’. Using MIIV-2SLS to model this relationship, we observed the predicted negative causal estimate. However, this result must be considered inconclusive owing to a wide confidence interval that included zero. We observed a negative causal estimate between measured fat mass and the brain latent that was similarly inconclusive owing to a wide confidence interval including zero. The fact that our study cohort comprised largely London university students facing minimal nutritional stress might help explain why our prediction of more readily observable somatic trade-offs in South Asian individuals did not find stronger support.

Nevertheless, the results presented here – using a novel method in MIIV-2SLS, and SEM more generally – represent an important first step in applying the ETH to a contemporary human cohort. The negative causal estimates we observed for brain and lean body tissues, and also brain and fat, are consistent with theory and prior evidence from some (but not all) studies of non-human animals. If accurately reflective of the strength of true biological relationships, we would have needed a larger sample to achieve a result with significance *p* < 0.05.

Aiello and Wheeler's ‘classic’ ETH predicted an evolutionary trade-off between the brain and a specific lean body tissue, the gut. This prediction relied on several lines of evidence, one of which considered relative constraints on the size of different tissues and organs in relation to body size, and the metabolic ‘expense’ of the tissues involved. Aiello and Wheeler (1995) theorised that investing additional energy in a costly organ like the brain required disinvestment in a similarly expensive tissue. Tissues may be considered more or less expensive according to their relative consumption of kilocalories per kilogram mass per day, with organs like the brain, gut, heart, kidneys and liver demonstrating higher mass-specific daily energy turnover than skeletal muscle, and even more so compared with adipose (fat) tissue (Elia, 1992; Shirley et al., 2019). Subsequent tests of Aiello and Wheeler's hypothesis have interrogated relationships between brain size and body tissues demonstrating both high and low energy turnover. These studies have been carried out in a range of non-human animals, both within and across species, and using experimental and observational approaches.

Some studies found support for the classic ETH (Kaufman, 2003; Kotrschal et al., 2013; Liao et al., 2016; Tsuboi et al., 2015), for example guppies experimentally selected for large brain size relative to body length demonstrated a concomitant decrease in gut size (Kotrschal et al., 2013). Evidence supporting a gut–brain trade-off has also been documented within and across other species of fish (Kaufman, 2003; Tsuboi et al., 2015) and frogs and toads (Liao et al., 2016), but not across bird (Isler & van Schaik, 2006), bat (Jones & MacLarnon, 2004) or mammal species (Navarrete et al., 2011). In a large sample of mammals including non-human primates, Navarrete et al. (2011) tested associations between an estimate of brain mass derived from cranial capacity and the gut, heart, kidneys, liver, lungs and spleen, as well as less metabolically expensive adipose tissue. Although the authors found little evidence for trade-offs between their brain mass proxy measure and any expensive tissues, the former was negatively related to adipose tissue, which is consistent with our data suggesting a negative link between brain and fat in humans. These results align with the theory that fat stores and brain tissue represent alternative strategies for buffering environmental stressors (Navarrete et al., 2011; Wells, 2012, 2016). Despite being both relatively encephalised and relatively fat mammals, hominins may plausibly have confronted energy allocation ‘decisions’ between a powerful central cognitive system capable of responding rapidly to short-term challenges, and a relatively cheap peripheral storage system designed to protect against longer-term threats to energy supply (Wells, 2012).

Beyond internal organs and fat, another tissue that has gained interest in the literature on energetics and human brain evolution is skeletal muscle. In humans, skeletal muscle consumes more energy per unit mass than adipose tissue (13 kcal per kg per day vs. 4.5 kcal), but less than the brain (240 kcal) and internal organs (i.e. the heart and kidneys consume ~440 kcal/kg/day) (Elia, 1992; Shirley et al., 2019). The relatively low value for skeletal muscle led Aiello and Wheeler to suggest that the human brain was unlikely to have been funded by a decrease in muscle mass (Aiello & Wheeler, 1995). Others, however, have disagreed, noting that humans have low levels of skeletal muscle relative to similarly sized primates (Leonard et al., 2003; Muchlinski et al., 2018), and that beyond resting requirements, the energy expenditure of muscle can increase up to a 100-fold during physical activity (Snodgrass et al., 2009). Moreover, the proportional contribution of skeletal muscle mass to total body mass in humans is considerably greater than that of the brain (~30–40 vs. ~2%) (Clarke & Sokoloff, 1999; Janssen et al., 2000) so that the overall percentage contribution of brain and skeletal muscle to total resting energy expenditure is in fact similar. Notably, Muchlinski et al. (2018) reported a negative correlation between endocranial volume and muscle mass across 10 primate species, although their sample did not include humans. Our results in humans are consistent with their findings.

Previous across-species tests of the ETH carried out in non-human animals garnered support for a genetic basis to trade-offs (Isler & van Schaik, 2006; Navarrete et al., 2011). There is also precedent for testing somatic trade-offs within species. For example, Warren and Iglesias (2012) investigated potential energetic constraints in investment among brain, liver and testes size in the coral reef fish *Thalassoma bifasciatum*, although they found no evidence for such constraints. Kaufman (2003), in contrast, found support for a trade-off between brain mass and stomach/intestines mass in the highly encephalised African freshwater fish *Gnathonemus petersii*. However, as noted above, the challenge of within-species tests of the ETH is that genetic vs. plastic, developmental and/or epigenetic effects on tissue size and covariation are not easily distinguished. Indeed, looking beyond evolutionary trends, evidence for short-term and developmental tissue trade-offs has been documented in humans. In the face of severe energy deficits resulting from famine conditions, for example, brain tissue has been shown to be preserved relative to skeletal muscle and fat mass (Rivers, 1988). On a similar basis, maternal constraints or poor nutritional supply in early life may lead to unequal energetic investment among the brain, organs and other body tissues of offspring (Hales & Barker, 1992; Latini et al., 2004; Yajnik et al., 2003). Interestingly, a study in athletes demonstrated evidence for an acute trade-off between mental and physical performance (with the former buffered at the expense of the latter) when brain and muscle function were forced into competition by experimental design (Longman et al., 2017).

The current study was not able to overcome the considerable challenge inherent to studying trade-offs within species, and we acknowledge, furthermore, that trade-offs observed within individuals, or within species, may not hold across species (as recently reviewed by Agrawal, 2020). (Although notably, trait associations expected to be equally strong across scales are those ‘related to body size, such as the allometric relationships between skeletal parameters, organ size and shape, and morphologies’; Agrawal, 2020: 14.)

At the same time, the fact that the brain–body tissue trade-offs suggested by our data may have arisen via life-course plasticity does not preclude the possibility that they represent evolved aspects of human body composition. As reviewed, relevant literature evidences brain–body tissue trade-offs operating at multiple timescales: evolutionary (Aiello & Wheeler, 1995; Kotrschal et al., 2013; Leonard et al., 2003; Muchlinski et al., 2018; Navarrete et al., 2011; Snodgrass et al., 2009; Wells, 2012), life-course/developmental (Hales & Barker, 1992; Latini et al., 2004; Rivers, 1988; Yajnik et al., 2003) and on a more immediate, acute basis (Longman et al., 2017). Furthermore, if somatic trade-offs arising via developmental plasticity were to persist across multiple generations and influence exposure to selective pressures, organ-size ratios could potentially reach new optima through genetic change (West-Eberhard, 1989). It is likely that any long-term physiological or biological trade-off that was eventually fixed genetically in hominins first arose through plasticity as organs and tissues competed for energy resources within life-course development. Relatedly, in their paper on brain–body allometry and mammalian brain size evolution, Smaers et al. (2021) note that ontogenetic and population-level (static) allometries shape evolutionary allometries.

Considered alongside prior findings, our results suggest that somatic trade-offs involving the brain and relatively expensive lean body tissues, as well as relatively inexpensive fat mass, may have been ‘options’ for funding an increasingly large and costly brain within the constraints of the hominin energy budget. Moreover, it may be possible for trade-offs amongst both low- and high-metabolic rate tissues to occur simultaneously. Our cohort was made up of healthy adults, thus the observed relationships, although tentative, arguably represent viable somatic energy allocation scenarios. We must, however, entertain the following possibility: even if the negative estimates we observed are true effects, the somatic trade-offs present in modern humans may not have played a role in the evolution of large human brains as hypothesised by Aiello and Wheeler.

Our inability to tease apart contributions and interactions of genetic and non-genetic effects in our test of somatic trade-offs is a considerable limitation of the current study. This and the relatively small sample size – which probably contributed to our lack of more precise estimates – nevertheless points to potentially fruitful next steps to test the ETH's prediction of tissue competition in humans. Namely, a future study will require a sample size with the power to capture small to medium effect sizes, and should also employ methods to capture the contribution of genetic variation to brain–body composition trade-offs. Possible avenues for testing this are using pedigree data or genome-wide association studies to examine genetic correlations, or with a twin study design, where variability associated with genetics and the environment can be controlled (e.g. Loos et al., 2002). Similarly in need of further research is the role of epigenetic modifications in shaping human body composition phenotypes, which would build on results from mouse models showing that epigenetic changes are associated with differential tissue growth across generations (Hardikar et al., 2015). These and other lines of evidence will be required to build a clearer picture of evolved somatic trade-offs within the human species.

Researchers interested in body composition and its correlates are investigating complementary issues of brain/body relationships; for example, how brain, body mass and metabolism associate with longevity (Peters et al., 2010), how acute stress impacts brain energy demand (Hitze et al., 2010) and how components of fat-free mass associate with fasting hunger (Casanova et al., 2022). Future studies may benefit from incorporating the concept of functional body composition into tissue trade-off models (Muller, 2013). This concept foregrounds the interrelationships among body components and functional outcomes such as energy expenditure and glucose and lipid metabolism, thereby going beyond organ/tissue size to define regulatory systems with clear implications for metabolic health (Muller et al., 2019). In the current study we have looked in new detail at organ/tissue correlations, but it is a limitation of our dataset that we were unable to extend this to evaluate functional and metabolic implications. A future analysis might also seek to test a hierarchical model that more explicitly situates body components in relation to the brain, e.g. centring the liver as the organ primarily responsible for supplying the brain with glucose (Muller et al., 2019). As an important methodological consideration, studies that seek to investigate functional body composition in the context of evolutionary trade-offs or replicate/extend the current analyses must specify the ‘level’ of body composition assessment (Wang et al., 1992), as there are differences to, for example, estimates of fat mass vs. adipose tissue (Hubers et al., 2019).

Despite the acknowledged limitations, our study took an important step in being the first to test a version of the ETH in humans, with two principal strengths. First, we used high-quality, criterion methods to measure volumes of the brain, internal organs, skeletal muscle and fat mass *in vivo*. Second, we went beyond commonly employed regression analyses to apply novel analytical methods to the question of somatic trade-offs. Utilising MIIVs in a SEM framework allowed us to (1) model what we believe to be the true outcomes of interest when testing hypotheses of inter-tissue competition, namely, nutritional resources invested in the organs or tissues of interest, and (2) better account for the key biases introduced with the use of observational data to improve causal inference. The importance of the latter point was highlighted when comparing our results with the results from traditional latent variable modelling, which showed a weak, positive effect of investment in brain on investment in body. Indeed, a recent meta-analysis found positive, rather than the hypothesised negative, genetic correlations among several life history traits, potentially owing to a lack of accounting for endogeneity (Chang et al., 2024). Overall, we hope that this work will stimulate further tests of the ETH's prediction of tissue competition in humans, and also the more general use of causal modelling approaches to test evolutionary questions.

## Supporting information

Shirley Bezerra et al. supplementary material Shirley Bezerra et al. supplementary material

## Supplementary material

To view supplementary material for this article, please visit https://doi.org/10.1017/ehs.2024.26

## Research transparency and reproducibility

Data will be made available to researchers upon request for the purpose of replicating the analyses or initiating collaboration to undertake further analyses.
