# Supplementary material for: Testing the expensive-tissue hypothesis’ prediction of inter-tissue competition using causal modelling with latent variables
Source: Evol Hum Sci. 2024 Oct 14;6:e33. doi: 10.1017/ehs.2024.26 (PMC11514623; doi:10.1017/ehs.2024.26)
Supplement: Shirley Bezerra et al. supplementary material [file S2513843X24000264sup001.docx]

**Supplementary Table S1**. MIIV-2SLS estimates for latent variable and measurement models testing negative causal effects of the latent variable “nutritional investment in brain tissues” on the latent “nutritional investment in lean body tissues,” and of the brain latent on measured fat mass using complete cases (n=67).

| **Measurement model** | Coefficients: | |  |  | Sargan test^2^: | | |
| --- | --- | --- | --- | --- | --- | --- | --- |
|  | Estimate | Std.Err^1^ | Lower CI | Upper CI | Statistic | df | *p* |
| Body measured by:  Organs  Skeletal muscle | 1.00  0.61 | 0.07 | 0.47 | 0.76 | 1.16 | 2 | 1.00 |
| Brain measured by:  Intracranial volume  Cerebrum  Cerebellum | 1.00  5.82  0.35 | 0.88  0.13 | 3.67  0.11 | 7.24  0.63 | 5.24  2.19 | 2  2 | 0.36  1.00 |
| **Latent variable model** |  |  |  |  |  |  |  |
| Body regressed on:  Height  Brain | 2.73  -0.27 | 0.52  0.35 | 1.72  -0.99 | 3.80  0.37 | 0.002 | 1 | 1.00 |
| Brain regressed on:  Height | 0.56 | 0.17 | 0.22 | 0.89 |  |  |  |
| Fat mass regressed on:  Height  Brain | 0.84  -0.29 | 1.38  1.24 | -1.74  -2.36 | 3.64  2.57 | 1.97 | 1 | 0.64 |
| Intercepts:  Body  Brain  Cerebellum  Cerebrum  Fat mass  Skeletal muscle | -20.09  3.94  8.22  11.47  10.53  2.91 | 7.81  2.74  1.70  11.31  20.27  1.47 | -34.19  -1.51  4.60  -6.83  -30.44  -0.10 | -3.13  9.43  11.35  38.87  49.61  5.75 |  |  |  |

^1^Bootstrapped standard errors based on 5000 repetitions

^2^Sargan tests for latent variable and measurement model equations test hypothesis that MIIVs are

uncorrelated with equation error, p>0.05 indicates failure to reject the null hypothesis of no correlation

CI, confidence interval

**Supplementary Table S2**. Traditional SEM maximum likelihood estimates^1^ for latent variable and measurement models testing effects of the latent variable “nutritional investment in brain tissues” on the latent “nutritional investment in lean body tissues,” and of the brain latent on measured fat mass (n=70).

| **Measurement model** |  | |  |  |
| --- | --- | --- | --- | --- |
|  | Estimate | Standard Error | Lower CI | Upper CI |
| Body measured by:  Organs  Skeletal muscle | 1.00  0.65 | 0.10 | 0.45 | 0.85 |
| Brain measured by:  Intracranial volume  Cerebrum  Cerebellum | 1.00  6.16  0.48 | 0.42  0.12 | 5.34  0.24 | 6.98  0.72 |
| **Latent variable model** |  |  |  |  |
| Body regressed on:  Height  Brain | 2.47  0.13 | 0.55  0.33 | 1.39  -0.52 | 3.55  0.78 |
| Brain regressed on:  Height | 0.55 | 0.17 | 0.22 | 0.88 |
| Fat mass regressed on:  Height  Brain | 1.24  -0.64 | 1.29  0.83 | -1.29  -2.27 | 3.77  0.99 |
| Intercepts:  Organs  Skeletal muscle  Intracranial volume  Cerebrum  Cerebellum  Fat mass | -20.54  -11.27  4.11  32.42  8.52  5.90 | 8.30  5.52  2.81  17.68  1.69  19.58 | -36.81  -22.09  -1.40  -2.23  5.21  -32.48 | -4.27  -0.45  9.62  67.07  11.83  44.28 |
| Variances:  Intracranial volume^2^  Organs  Skeletal muscle  Cerebrum  Cerebellum  Fat mass  Body  Brain | 0.000  1.95  1.11  12.52  0.97  43.22  5.66  0.90 | 1.17  0.51  2.12  0.16  7.31  1.47  0.15 | -0.34  0.11  8.36  0.66  28.89  2.78  0.61 | 4.24  2.11  16.68  1.28  57.53  8.54  1.19 |

^1^Set missing = “fiml” within the sem function of R package lavaan; no other arguments were specified

^2^Value fixed to zero

CI, confidence interval
